# Residual Inflammation Indicated by High-Sensitivity C-Reactive Protein Predicts Worse Long-Term Clinical Outcomes in Japanese Patients after Percutaneous Coronary Intervention

**DOI:** 10.3390/jcm9041033

**Published:** 2020-04-06

**Authors:** Norihito Takahashi, Tomotaka Dohi, Hirohisa Endo, Takehiro Funamizu, Hideki Wada, Shinichiro Doi, Yoshiteru Kato, Manabu Ogita, Iwao Okai, Hiroshi Iwata, Shinya Okazaki, Kikuo Isoda, Katsumi Miyauchi, Kazunori Shimada

**Affiliations:** 1 Department of Cardiovascular Medicine, Juntendo University Graduate School of Medicine, 2-1-1 Hongo, Bunkyo-ku, Tokyo 113-0033, Japan; n-takaha@juntendo.ac.jp (N.T.);; 2 Department of Cardiology, Juntendo University Shizuoka Hospital, 1129 nagaoka, Izunokuni-shi 410-2295, Shizuoka, Japan

**Keywords:** hs-CRP, residual risk, biomarker, inflammation, coronary artery disease, percutaneous coronary intervention

## Abstract

The aim of this study was to investigate the long-term clinical impact of residual inflammatory risk (RIR) by evaluating serial high-sensitivity C-reactive protein (hs-CRP) in Asian patients with coronary artery disease (CAD). We evaluated 2032 patients with stable CAD undergoing percutaneous coronary intervention (PCI) with serial hs-CRP measurements (2 measurements, 6–9 months apart) from the period 2000 to 2016. A high-RIR was defined as hs-CRP > 0.9 mg/L according to the median value. Patients were assigned to four groups: persistent-high-RIR, increased-RIR, attenuated-RIR, or persistent-low-RIR. Major adverse cardiac events (MACE) and all-cause death were evaluated. MACE rates in patients with persistent high, increased and attenuated RIR were significantly higher than in patients with persistent low RIR (*p* < 0.001). Moreover, the rate of all-cause death was significantly higher among patients with persistent high and increased RIR than among patients with attenuated and persistent low RIR (*p* < 0.001). After adjustment, the presence of persistent high RIR (hazard ratio (HR) 2.22; 95% confidence interval (CI) 1.37–3.67, *p* = 0.001), increased RIR (HR 2.25, 95%CI 1.09–4.37, *p* = 0.029), and attenuated RIR (HR 1.94, 95%CI 1.14–3.32, *p* = 0.015) were predictive for MACE. In addition, presence of persistent high RIR (HR 2.07, 95%CI 1.41–3.08, *p* < 0.001) and increased RIR (HR 1.94, 95%CI 1.07–3.36, *p* = 0.029) were predictive for all-cause death. A high RIR was significantly associated with MACE and all-cause death among Japanese CAD patients. An evaluation of changes in inflammation may carry important prognostic information and may guide the therapeutic approach.

## 1. Introduction

Coronary artery disease (CAD) is one of the leading causes of death [1], and the World Health Organization reported that about 9 million people died from ischemic heart disease in 2016 [2]. With the advent of lipid-lowering therapies represented by statins, cardiovascular events after percutaneous coronary intervention have significantly reduced. However, even with intensive lipid-lowering therapy, cardiovascular death (CVD) risk persists over long-term follow-up [3,4,5].

Inflammation plays an important role in promoting endothelial cell damage and the progression of atherosclerosis, which triggers coronary artery disease and adverse cardiovascular events [6,7]. Among several inflammation parameters, the C-reactive protein (CRP) is widely considered a valuable predictor of CAD [8]. There have been many reports on the association between CRP values and adverse clinical outcome, and CRP has been established as an independent predictor of cardiovascular events [9,10,11,12]. However, CRP concentrations vary in ethnic groups, with especially lower levels documented in Asian populations [13,14]. Recently, residual inflammatory risk (RIR) has been considered more important than before, because interventions to address inflammation could reduce CVD risk [15]. Kalkman et al. showed that patients with persistent high RIR had higher all-cause mortality in patients undergoing percutaneous coronary intervention (PCI) at 1-year follow-up [16]. However, the long-term clinical impact of different patterns of RIR in stable CAD patients after PCI remains unclear for Asian populations. 

We thus aimed to evaluate the associations of RIR with long-term adverse cardiovascular events and mortality in Japanese patients who underwent PCI.

## 2. Methods and Patients

### 2.1. Study Design and Subjects

**Figure 1:**
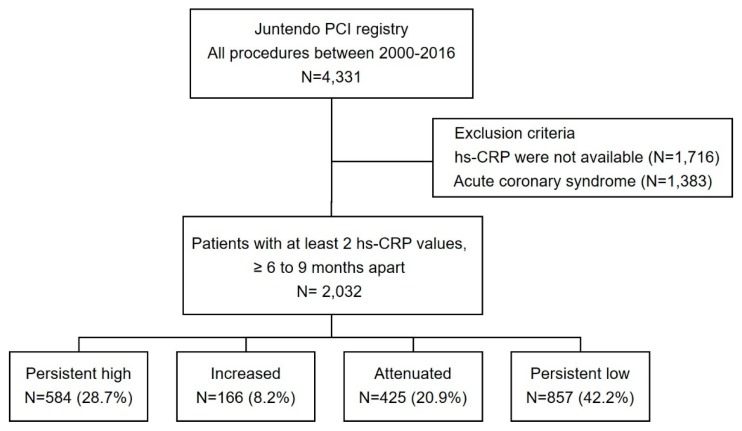
Study flow chart. PCI, percutaneous coronary intervention; hs-CRP, high-sensitivity C-reactive protein; ACS, acute coronary syndrome; RIR, residual inflammatory risk.

The current investigation was a single-center, observational, retrospective cohort study at Juntendo University Hospital (Tokyo, Japan). Among the consecutive patients with CAD who underwent PCI in our institution from January 2000 to December 2016, we enrolled those patients for whom two hs-CRP measurements were made at an interval of 6–9 months (Figure 1). Exclusion criteria were as follows: (1) patients with no hs-CRP data or only one measurement of hs-CRP; or (2) patients who developed acute coronary syndrome (ACS). The study cohort was divided into four groups according to RIR. High RIR was defined as hs-CRP > 0.9 mg/L according to the median value. The groups were defined as: persistent high RIR (high hs-CRP on both measurements); increased RIR (low hs-CRP on first measurement, high hs-CRP on second measurement); attenuated RIR (high hs-CRP on first measurement, low hs-CRP on second measurement); or persistent low RIR (low hs-CRP on both measurements). We performed an additional analysis to compare patient characteristics of the included group and excluded group (Supplementary Figure S1, Supplementary Table S1). The study protocols were approved by the Ethics Committee of Juntendo University Hospital, Tokyo, Japan and conducted in accordance with the principles of the Declaration of Helsinki. 

### 2.2. Data Collection

Demographic data, information on coronary risk factors, comorbidities and medications were collected from the institutional database. Blood samples were collected during the early morning of index PCI after an overnight fast. Patients with blood pressure ≥ 140/90 mmHg or under antihypertensive drugs were regarded as hypertensive. We defined diabetes mellitus as either hemoglobin (Hb)A1c ≥ 6.5%, medication with oral hypoglycemic drugs, or insulin injections. We defined chronic kidney disease (CKD) as an estimated glomerular filtration rate (eGFR) < 60 mL/min/1.73 m^2^, and calculated eGFR using the diet in renal disease equation modified with a Japanese coefficient using baseline serum creatinine. Current smoker status was defined as an individual who was a smoker at the time of PCI or had quit smoking within 1 year before PCI. Follow-up blood samples were collected in the early morning of follow-up angiography. Serum hs-CRP was measured at pre-index PCI and pre-follow up angiography using high sensitivity latex turbidimetric immunoassay. Median time from index PCI to follow up angiography was 8.1 months (interquartile range (IQR), 6.7–8.6 months). Mortality data and information for adverse events were collected by serial contact with the patients or their families and by the medical records and were obtained response to questionnaires sent to patients or their families and telephone contact. All data were collected by blinded investigators.

### 2.3. Study Endpoints

The endpoints of this study were all-cause death and major adverse cardiac events (MACE), defined as a composite of cardiovascular death, non-fatal myocardial infarction (MI) and non-fatal cerebral infarction (CI). Survival data and information about the incident were obtained by serial contact with patients and were assessed from the medical records of patients. Mortality data were collected from the families of patients who died at home, and details of events associated with cause of death were supplied by other hospitals or clinics to which the patient had been admitted. All data were collected by blinded investigators. Cardiovascular death was defined as death caused by myocardial infarction, heart failure, or sudden death. Time to event was measured from the second hs-CRP measurement.

### 2.4. Statistical Analysis

Categorical data are presented as numbers and percentages and compared using the chi-square test. Continuous variables are expressed as mean ± standard deviation or as median and interquartile range and were compared using one-way analysis of variance or the Kruskal–Wallis test. We applied analysis of variance (ANOVA) to persistent high RIR, increased RIR, attenuated RIR and persistent low RIR, then post-hoc pairwise comparisons between each group were conducted with Bonferroni corrections. Unadjusted cumulative event rates were estimated using Kaplan–Meier curves and were compared among the four groups. Multivariate analysis included clinically important variables, such as age, sex, hypertension, chronic kidney disease (CKD), diabetes mellitus (DM), dyslipidemia (DL), body mass index (BMI), smoking status, multivessel disease, left ventricular ejection fraction (LVEF), low-density lipoprotein cholesterol (LDL-C), high-density lipoprotein cholesterol (HDL-C), triglycerides (TG), use of statins and RIR. Values of *p* < 0.05 were considered to indicate statistical significance, unless otherwise indicated. All data were analyzed using JMP for Windows version 12.0 (SAS Institute, Cary, NC, USA).

## 3. Results

### 3.1. Study Population

Figure 1 shows the flow chart for the study population. Of the 4331 patients who underwent PCI, patients for whom hs-CRP data at both time of PCI and follow-up were unavailable (*n* = 1716) or who showed ACS (*n* = 1383) were excluded. Finally, 2032 patients were enrolled and divided into four groups according to RIR. Persistent high RIR was seen in 584 patients (28.7%), increased RIR in 166 patients (8.2%), attenuated RIR in 425 patients (20.9%), and persistent low RIR in 857 (42.2%).

### 3.2. Baseline Characteristics

Table 1 shows the baseline characteristics of patients. Mean age in the total cohort was 66.6 ± 9.7 years old. No significant differences were seen in age, sex, dyslipidemia or multivessel disease. Patients with persistent high RIR exhibited a higher prevalence of chronic kidney disease, higher body mass index, higher concentrations of TG and LDL-C, and a higher frequency of current smoker status.

### 3.3. Clinical Outocomes

**Figure 2:**
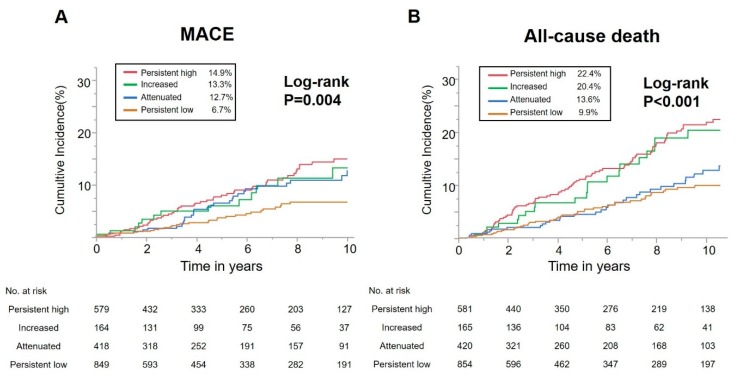
Kaplan–Meier curve for major adverse cardiovascular events (MACE) and all-cause death. (**A**) Long-term major adverse cardiovascular events (MACE) (composite endpoint defined as cardiovascular death, non-fatal myocardial infarction, or non-fatal cerebral infarction). (**B**) Long-term all-cause mortality.

The median duration of follow-up was 4.9 years (IQR, 1.7–9.6 years) and prognostic data were fully documented during the entire follow-up period. During follow-up, 179 (15.6%) all-cause deaths and 124 (11.1%) MACE occurred, including 53 (4.7%) cardiovascular deaths, 25 (2.3%) non-fatal MIs, and 50 (4.7%) non-fatal CIs. Figure 2 shows cumulative incidence curves for MACE and all-cause death. Rates of MACE were significantly higher in patients with persistent high, increased or attenuated RIR than in patients with persistent low RIR (*p* = 0.004). In addition, rates of all-cause death were significantly higher in patients with persistent high or increased RIR than in patients with attenuated or persistent low RIR (*p* < 0.000). 

Furthermore, we performed additional Kaplan-Meier analysis excluding the patients with impaired renal function as a confounding factor. As a result, incidences of MACE were also significantly higher in patients with persistent high, increased or attenuated RIR than in patients with persistent low RIR (*p* = 0.014). In addition, incidences of all-cause death were significantly higher in patients with persistent high or increased RIR than in patients with attenuated or persistent low RIR (*p* < 0.001) (Supplementary Figure S2).

### 3.4. Association between RIR and MACE and All-Cause Death

Table 2 shows the results of Cox proportional hazard regression analysis for MACE and all-cause death. Patients with persistent high RIR, increased RIR, and attenuated RIR showed a significantly higher frequency of MACE than patients with persistent low RIR (hazard ratio (HR) 2.22; 95% confidence interval (CI) 1.37–3.67, *p* = 0.001; HR 2.25, 95%CI 1.09–4.37, *p* = 0.029, respectively; HR 1.94, 95%CI 1.14–3.32, *p* = 0.015), even after adjusting for other risk factors. Persistent high RIR and increased RIR had significantly higher frequencies of all-cause death than persistent low RIR (HR 2.07, 95%CI 1.41–3.08, *p* < 0.001; HR 1.94, 95%CI 1.07–3.36, *p* = 0.029, respectively). No significant association with all-cause death was seen for attenuated RIR or persistent low RIR.

## 4. Discussion

The major findings of the current study were as follows: (1) median hs-CRP in this cohort was 0.9 mg/L; (2) patients with persistent high RIR showed a significantly higher frequency of MACE and all-cause death than those with persistent low RIR; and (3) even after adjusting for clinically important covariates, persistent high RIR was associated with poorer long-term clinical outcomes.

In this study, we showed the clinical significance of serial inflammatory status assessment among East Asian stable CAD patients undergoing PCI with long-term follow up. Previous studies have shown that inflammation plays a key role in CAD and triggers atherosclerosis. Immune cells dominate early atherosclerotic lesions, their effector molecules drive the progression of these lesions, and activation of inflammation can trigger ACS [6,17,18,19,20]. As a well-known parameter of inflammation, CRP is also considered a valuable predictor of CAD risk [8]. The measurement of hs-CRP has been proposed to identify individuals at a high risk of cardiovascular events [21,22,23,24]. However, those studies only assessed single measurements of hs-CRP. Mani et al. reported initial and subsequent increases in hs-CRP levels after acute coronary syndrome were associated with adverse cardiovascular events [25]. However, the prognostic impact of serial hs-CRP measurement on long-term clinical outcomes in Asian stable CAD patients remained unclear.

Median hs-CRP level at index PCI was 0.9 mg/L in the current study, much lower than values previously reported from Western countries [26,27,28,29]. In the field of primary prevention among the Japanese population, hs-CRP values have been reported as lower than in Western populations [13,30]. The highest values have commonly been found in African-American populations, followed by Hispanic, South Asian, Caucasian, and East Asian populations [31,32]. Many differences are seen in not only genetic factors, but also food and lifestyle between Asians (including Japanese) and Westerners, which might affect hs-CRP levels. 

Statin therapy targeting LDL-C markedly reduces CVD events in both primary and secondary prevention. Although therapies to strongly reduce LDL-C have been shown to prevent CVD events, eliminating all CVD events is not possible, leaving a “residual risk” [3,4,5]. Of the secondary prevention patients in the Pravastatin or Atorvastatin Evaluation and Infection Therapy-Thrombolysis in Myocardial Infarction 22 (PROVE-IT) trial treated with aggressive statin therapy, those who achieved LDL-C levels < 70 mg/dL and hs-CRP levels < 2 mg/dL displayed a lower incidence of adverse vascular events compared with those who achieved only one or neither of these independent treatment targets [12]. The Further Cardiovascular Outcomes Research with PCSK9 inhibition in Patients with Elevated Risk (FOURIER) trial demonstrated that inflammatory status is strongly associated with adverse clinical outcomes even when LDL-C is markedly reduced by PCSK9 inhibitor [33]. The beneficial effects of statins include not only lowering LDL-C levels, but also anti-inflammatory effects [34]. The current study demonstrated that about half of patients exhibited RIR at follow-up despite statin treatment. For these populations in whom cholesterol is no longer the primary problem, RIR has recently been reported to remain [35]. Such patients might benefit from anti-inflammatory treatments over additional lipid lowering [5]. 

Although a high inflammation status has been associated with increased cardiovascular events, whether intervention is warranted for patients with elevated CRP remains controversial. In the Canakinumab Antiinflammatory Thrombosis Outcome Study (CANTOS) trial, canakinumab reduced inflammation and adverse cardiac events in patients with prior MI and increased baseline hs-CRP ≥ 2mg/L, without interfering with lipid levels [15]. Furthermore, the magnitude of hs-CRP reduction following a single dose of canakinumab was associated with favorable clinical outcome. That finding suggests that a lower hs-CRP is better for inflammation reduction with canakinumab [36]. Moreover, the efficacy of other inflammation-reducing therapies has been reported. In the COLchicine Cardiovascular Outcomes Trial in Coronary Disease (COLCOT), the effect of colchicine on cardiovascular events was investigated in patients after MI. The colchicine group showed a 23% lower risk than the placebo group [37]. Conversely, the Colchicine in percutaneous coronary intervention (COLCHICINE-PCI) trial recently demonstrated that pre-PCI colchicine reduced post-PCI inflammation, but did not reduce the risk of PCI-related myocardial injury or adverse cardiac events (NCT02594111). The Cardiovascular Inflammation Reduction Trial (CIRT) reported that low-dose methotrexate did not reduce CRP levels and did not result in fewer cardiovascular events than placebo among stable CAD patients [38]. The effects of bromodomain and extra-terminal protein inhibition with apabetalone on cardiovascular outcomes in patients with acute coronary syndrome and diabetes (BETonMACE) trial did not reduce rates of cardiovascular death, non-fatal myocardial infarction or non-fatal stroke among diabetic patients after acute coronary syndrome [39] (NCT02586155). The present study showed that hs-CRP was reduced in about half of patients at 6–9 months after index PCI. In these trials, although differences exist between drugs, inflammation may have reduced spontaneously as well due to the effects of specific drugs. 

This study had several limitations that require consideration. First, because this was a single-center, retrospective, observational study, unknown confounding factors might have affected outcomes despite analytical adjustments. Second, relatively few events occurred, resulting in a lack of significant differences in outcome. Third, although we generally reschedule or discontinue PCI when patients are being treated for active infection or malignant disease, some patients with active infections, hidden malignancies or autoimmune disease might have been included. Finally, because we measured the second hs-CRP value at the time of follow-up angiogram, patients who did not perform follow-up angiogram were excluded. As shown in the Supplementary Figure S1, 37 patients died by 9 months in patients who underwent PCI for stable angina. In addition, 512 patients did not perform follow-up angiogram, 647 patients did not measure hs-CRP for the second time, and a total of 647 patients were excluded from the analysis. Clinical characteristics of the patients who were included and excluded in this study were shown Supplementary Table S1. There were no significant differences between the two groups for age, gender, hypertension, dyslipidemia, diabetes, multivessel disease, and LVEF. However, the excluded group had a higher proportion of patients with renal dysfunction. We generally plan to perform a follow-up angiogram if the patient’s consent is obtained, but the follow-up angiogram might have been omitted in a group with impaired renal function. However, additional analysis has also shown that RIR is an independent predictor in excluded patients with impaired renal function (Supplementary Figure S2). 

## 5. Conclusions

High RIR was significantly associated with MACE and all-cause death among Asian patients after PCI. An evaluation of changes in inflammation may carry important prognostic information and guide the therapeutic approach.

## Figures and Tables

**Table 1:** Baseline Characteristics of the Study Population.

	Overall (N = 2032)	Persistent High (n = 584)	Increased (n = 166)	Attenuated (n = 425)	Persistent Low (n = 857)	*p*
Age, years	66.6 ± 9.7	67.3 ± 0.4	66.1 ± 0.8	66.1 ± 0.8	66.4 ± 0.3	0.19
Male, n (%)	1688 (83.0)	482 (82.5)	139 (83.7)	366 (86.1)	701 (81.8)	0.25
Hypertension, n (%)	1485 (73.1)	447 (76.5)	115 (69.3)	318 (74.8)	605 (70.6)	0.043
CKD, n (%)	483 (23.7)	170 (29.1)	40 (24.1)	118 (27.7)	155 (13.1)	<0.001
Dyslipidemia, n (%)	1555 (76.5)	451 (77.2)	129 (77.7)	326 (76.7)	649 (75.7)	0.90
Diabetes, n (%)	899 (44.2)	273 (46.8)	86 (51.8)	189 (44.8)	351 (41.0)	0.028
Current smoking, n (%)	452 (22.2)	158 (27.1)	40 (24.1)	94 (22.1)	160 (18.7)	0.002
Family history of CAD, n (%)	596 (29.4)	167 (28.6)	43 (25.9)	109 (25.7)	277 (32.3)	0.06
BMI, kg/m^2^	24.5 ± 3.3	25.0 ± 0.1	24.3 ± 0.3	24.6 ± 0.2	24.1 ± 0.1	<0.001
LVEF, %	62.7 ± 10.9	62.1 ± 0.5	63.6 ± 0.9	61.5 ± 0.6	63.4 ± 0.4	0.016
Multivessel disease, n (%)	1215 (60.3)	360 (62.0)	104 (63.0)	254 (60.1)	497 (58.6)	0.56
LMT lesion, n (%)	68 (3.3)	17 (2.9)	5 (3.0)	10 (2.4)	36 (4.2)	0.30
LAD	949 (46.7)	245 (42.0)	76 (45.8)	216 (50.8)	412 (48.1)	0.030
RCA	598 (29.4)	186 (31.9)	42 (25.3)	130 (30.6)	240 (28.0)	0.25
LCX	394 (19.4)	125 (21.4)	40 (24.1)	66 (15.5)	163 (19.0)	0.049
Medication						
Aspirin, n (%)	1962 (97.1)	566 (97.3)	160 (96.4)	405 (96.4)	830 (97.5)	0.67
β-blocker, n (%)	1047 (51.9)	297 (51.0)	78 (47.0)	229 (54.7)	443 (52.1)	0.38
CCB, n (%)	916 (45.4)	271 (46.7)	71 (42.8)	189 (45.1)	384 (45.2)	0.82
ACE/ARB, n (%)	973 (48.2)	300 (51.6)	76 (45.8)	208 (49.6)	389 (45.8)	0.13
Statin, n (%)	1507 (74.6)	395 (68.0)	129 (77.7)	311 (74.1)	672 (79.0)	<0.001
Baseline laboratory findings						
hs-CRP, mg/L	0.9 (0.4–2.2)	2.7 (1.5–5.7)	0.5 (0.3–0.7)	2.0 (0.1–0.4)	0.4 (0.2–0.5)	<0.001
Hemoglobin, g/dL	13.7 (12.5–14.7)	13.5 (12.0–14.7)	13.7 (12.5–14.9)	13.6 (12.4–14.6)	13.9 (12.8–14.7)	0.003
Serum creatinine, mg/dL	0.81 (0.69–0.94)	0.83 (0.71–0.96)	0.82 (0.72–0.95)	0.84 (0.73–0.97)	0.78 (0.66–0.91)	0.008
TG, mg/dL	121 (91–162)	133 (97–180)	115 (93–154)	118 (89–155)	115 (88–153)	<0.001
HDL-C, mg/dL	42 (36–52)	40 (33–48)	45 (36–53)	40 (35–47)	45 (38–54)	<0.001
LDL-C, mg/dL	99 (81–122)	104 (83–124)	97 (83–122)	100 (83–126)	95 (79–117)	0.001
Lp (a), mg/dL	18 (9–32.3)	18.1 (9.0–34.6)	17.0 (10.0–32.2)	20.9 (10.0–34.0)	16.0 (8.0–30.0)	0.16
BNP, pg/dL	36.6 (18.1–81.9)	38.8 (17.1–92.1)	34.2 (19.8–80.4)	41.4 (17.7–107.4)	33.9 (18.6–67.0)	0.001
Follow-up laboratory findings						
hs-CRP, mg/L	0.6 (0.3–1.5)	2.2 (1.4–4.0)	2.3 (1.4–5.3)	0.5 (0.3–0,7)	0.3 (0.2–0.5)	<0.001
LDL-C, mg/dL	89 (74–107)	93 (76–111)	90 (76–110)	90 (75–106)	89 (75–106)	0.001

CKD, chronic kidney disease; CAD, coronary artery disease; BMI, body mass index; TG, triglyceride; HDL-C, high-density lipoprotein-cholesterol; LDL, low-density lipoprotein-cholesterol; Lp (a), lipoprotein (a); BP, blood pressure; LVEF, left ventricular ejection fraction; ACS, acute coronary syndrome; LMT, left main trunk; BNP, b-type natriuretic peptide; CCB, calcium channel blocker; ACE, angiotensin-converting enzyme inhibitor; ARB, angiotensin II receptor blocker.

**Table 2:** Unadjusted and adjusted models for outcomes according to residual inflammatory risk.

		Unadjusted Model		Adjusted Model					
				Model 1		Model 2		Model 3	
Event	Inflammatory Status	HR (95% CI)	*p*	HR (95% CI)	*p*	HR (95% CI)	*p*	HR (95% CI)	*p*
All-cause death	Persistent high	2.34 (1.64–3.39)	<0.001	2.18 (1.53–3.16)	<0.001	2.03 (1.39–3.00)	<0.001	2.08 (1.41–3.11)	<0.001
	Increased	2.97 (1.21–3.42)	0.009	2.07 (1.21–3.42)	0.009	1.97 (1.09–3.40)	0.026	2.05 (1.13–3.11)	0.019
	Attenuated	1.22 (0.76–1.91)	0.40	1.22 (0.77–1.92)	0.40	1.30 (0.80–2.07)	0.28	1.37 (0.84–2.18)	0.20
MACE	Persistent high	2.30 (1.49–3.63)	<0.001	2.17 (1.40–3.44)	<0.001	2.33 (1.45–3.82)	<0.001	2.38 (1.46–3.96)	<0.001
	Increased	2.13 (1.10–3.92)	0.026	1.96 (0.99–3.66)	0.054	2.24 (1.09–4.34)	0.029	2.35 (1.14–4.58)	0.022
	Attenuated	1.82 (1.11–3.01)	0.019	1.76 (1.06–2.92)	0.028	1.94 (1.14–3.30)	0.015	2.00 (1.17–3.43)	0.012

Patients with persistent low residual inflammatory risk (RIR) were used as references. Covariates of the fully adjusted model are as below. Model 1: age, sex, RIR. Model 2: age, sex, RIR, hypertension (HT), chronic kidney disease (CKD), diabetes mellitus (DM), dyslipidemia (DL), body mass index (BMI), smoking status, multivessel disease (MVD), left ventricular ejection fraction (LVEF). Model 3: age, sex, RIR, HT, CKD, DM, DL, BMI, smoking status, MVD, LVEF, low-density lipoprotein-cholesterol, high-density lipoprotein–cholesterol, triglycerides, use of statins. HR, hazard ratio; 95%CI, 95% confidence interval.

## Supplementary Materials

The following are available online at https://www.mdpi.com/2077-0383/9/4/1033/s1, Figure S1: Study flow chart, Figure S2: Kaplan-Meier curve for MACE and all-cause death in patients with preserved renal function. (A) Long-term major adverse cardiovascular events (MACE) (composite endpoint defined as cardiovascular death, non-fatal myocardial infarction, or non-fatal cerebral infarction). (B)Long-term all-cause mortality, Table S1: Patients Characteristics.
