# Supplementary material for: Residual Inflammation Indicated by High-Sensitivity C-Reactive Protein Predicts Worse Long-Term Clinical Outcomes in Japanese Patients after Percutaneous Coronary Intervention
Source: J Clin Med. 2020 Apr 6;9(4):1033. doi: 10.3390/jcm9041033 (PMC7230848; doi:10.3390/jcm9041033)
Supplement: Supplementary file 1 [file jcm-09-01033-s001.pdf]

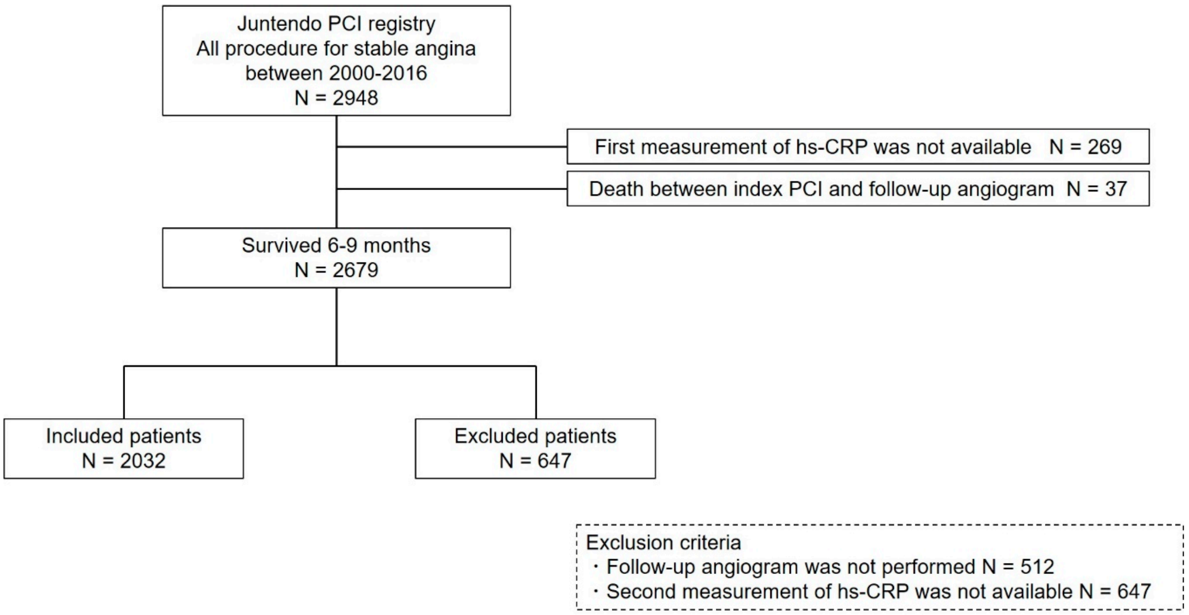

Supplementary Figure 1. Study flow chart. PCI, percutaneous coronary intervention; hs-CRP, high-sensitivity C-reactive protein; ACS, acute coronary syndrome; RIR, residual inflammatory risk.

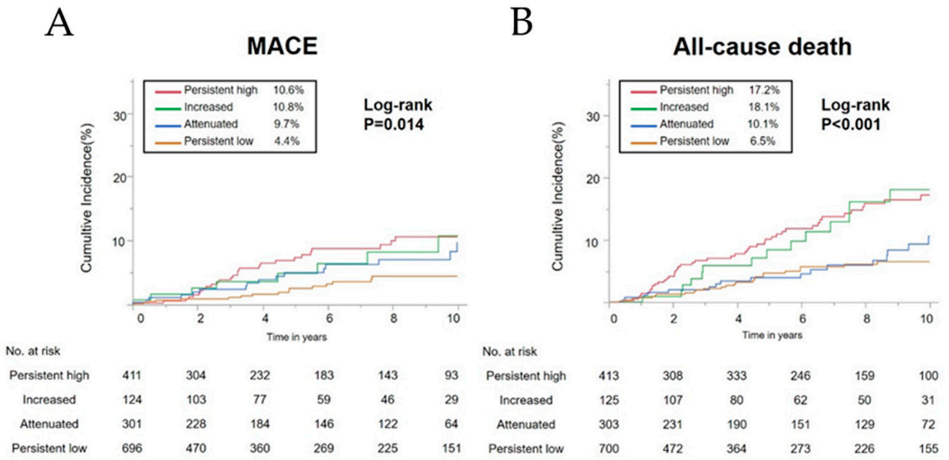

Supplementary Figure 2. Kaplan-Meier curve for MACE and all-cause death in patients with preserved renal function. (**A**) Long-term major adverse cardiovascular events (MACE) (composite endpoint defined as cardiovascular death, non-fatal myocardial infarction, or non-fatal cerebral infarction); (**B**)Long-term all-cause mortality.

Supplementary Table 1. Patients Characteristics.

	Included patients (n = 2032)	Excluded patients (n = 647)	p
Age, years	66.6 ± 9.7	67.3 ± 10.9	0.11
Male, n(%)	1688(83.1)	535 (82.7)	0.82
LVEF, %	62.7 ± 10.9	62.8 ± 12.9	0.82
Multivessel disease, n (%)	1215 (60.3)	393 (61.3)	0.65
Hypertension, n(%)	1458 (73.1)	471 (72.8)	0.89
CKD, n(%)	483 (23.8)	213 (32.9)	<0.001
Dyslipidemia, n(%)	1555 (76.5)	484 (74.8)	0.37
Diabetes, n(%)	899 (44.2)	290 (44.8)	0.80
eGFR	69.6 ± 17.8	66.1 ± 20.9	<0.001
BMI	24.5 ± 3.3	24.2 ± 3.3	0.08

LVEF, left ventricular ejection fraction; CKD, chronic kidney disease; eGFR, estimated glomerular filtration rate; BMI, body mass index.
